# An unusual case of *Acanthamoeba Polyphaga* and *Pseudomonas Aeruginosa* keratitis

**DOI:** 10.1186/1746-1596-9-105

**Published:** 2014-06-03

**Authors:** Jiaxu Hong, Jian Ji, Jianjiang Xu, Wenjun Cao, Zuguo Liu, Xinghuai Sun

**Affiliations:** 1 Department of Ophthalmology and Visual Science, Eye, Ear, Nose, and Throat Hospital, School of Shanghai Medicine, Fudan University, 83 Fenyang Road, Shanghai 200031, China; 2 Health Communication Institute, Fudan University, 130 Dongan Road, Shanghai 200032, China; 3 Eye Institute of Xiamen University, Fujian 361005, China; 4 Myopia Key Laboratory of Ministry of Health, Shanghai, China

**Keywords:** Contact lens, *Acanthamoeba* species, *Pseudomonas aeruginosa*

## Abstract

**Virtual Slides:**

The virtual slide(s) for this article can be found here: http://www.diagnosticpathology.diagnomx.eu/vs/5168343391150859

A 56-year-old woman with a history of disposable soft contact lens wear was referred to our university eye center for a corneal ulcer. Based on the microbial culture, the initial diagnosis was bacterial keratitis, which was unresponsive to topical fortified antibiotics. The patient was then examined using *in vivo* confocal microscopy, which revealed *Acanthamoeba* infection. This case emphasizes the need to suspect *Acanthamoeba* infection in soft contact lens wearers who present with progressive ulcerative keratitis or progressively worsening corneal ulcers that are not responsive to the usual antimicrobial therapy. It is also important to consider the possibility of a coinfection with bacterial and *Acanthamoeba* species.

## Background

*Acanthamoeba* keratitis (AK) is a destructive disease characterized by significant visual morbidity, and prompt diagnosis is important for a good visual outcome. Like AK, *Pseudomonas aeruginosa* keratitis usually progresses rapidly and presents with suppurative stromal infiltrate and marked mucopurulent exudate. It should be noted that there is a possibility, in theory, that AK can develop in eyes with advanced bacterial keratitis. Coinfections with other microorganisms have been reported in patients with culture-proven AK [1,2]. However, the exact clinical characteristics of such mixed infections remain unknown. Few publications have addressed this issue. Herein, we report an unusual case of coinfection with *Acanthamoeba polyphaga* and *Pseudomonas aeruginosa* as causes of corneal keratitis in a contact lens wearer in Shanghai.

## Case presentation

**Figure 1:**
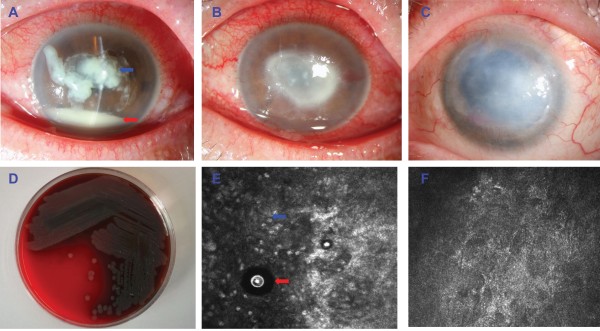
**An unusual keratitis case of coinfection with *Acanthamoeba polyphaga *and *Pseudomonas aeruginosa. *(A)** Slit-lamp microscopic image of the left eye showed severe central corneal infiltrate (blue arrow) with intensive conjunctival injection. The anterior chamber had 20% hypopyon (black arrow). **(B)** After 1 week of treatment with topical antibiotics, a large central corneal ulcer with an underlying grayish-white, paracentral, ring-shaped stromal infiltrate was identified. **(C)** After 12 months of treatment with topical antibiotics, the left eye displayed no signs of inflammation, but there were some superficial blood vessels in the peripheral cornea and a large, central corneal scar obscuring the visual axis. **(D)** Microbiological cultures obtained from a superficial corneal swab showed the presence of *Pseudomonas aeruginosa.I*. **(E)***In vivo* confocal microscopy examination showed the presence of oval to round, double-walled, highly refractile structures with a polygonal inner wall, varying 12–25 μm in size (red arrow), with infiltration of inflammatory cells (blue arrow). **(F)** The *Acanthamoeba* cysts could not be detected by IVCM examination after 12 months of treatment.

A 56-year-old female teacher presented with a two-week history of increasing pain and redness in the left eye. The patient had a five-year history of disposable soft contact lens wear and was sometimes careless with the disinfecting routine. Occasionally, she would rinse her contact lenses and case in tap water instead of a sterile saline solution. The patient stated that she had no history of ocular trauma, overnight contact lens wear, hypertension, or diabetes. She had no known drug allergies and no systemic infections at the time of her presentation. She had been treated one week previously for a *P. aeruginosa* corneal ulcer and had received topical fortified tobramycin and levofloxacin, to which the organism had shown sensitivity in the laboratory (Figures 1A and D). She denied any significant improvement of her symptoms and signs. On examination, her best-corrected visual acuities were counting fingers in the left eye and 20/20 in the right. She had a large central corneal ulcer with an underlying grayish-white, paracentral, ring-shaped stromal infiltrate (Figure 1B). The hypopyon in the anterior chamber had improved significantly after the initial treatment with the topical antibiotics. The right eye was normal. The patient was examined using an *in vivo* confocal microscopy (IVCM). Interestingly, the IVCM images showed the presence of oval to round, double-walled, highly refractile structures with a polygonal inner wall, varying 12–25 μm in size. The morphology was consistent with that of *Acanthamoeba* cysts reported in other articles [3,4]. The structures were surrounded by inflammatory cells (Figure 1E).

Upon confirmation of the presence of the *Acanthamoeba* species via the IVCM examination, an additional treatment regimen of topical 0.2% metronidazole and 0.02% polyhexamethylene biguanide drops given hourly was begun. Over the next two weeks, the stromal infiltrate showed slight improvement, and after 3-months treatment, the *Acanthamoeba* cysts could not be detected by IVCM examination. Five months after that, the patient underwent combined phacoemulsification and viscogoniosynechialysis due to the presence of peripheral synechiae and a cataract. The topical treatment was continued for a prolonged period, tapering off over 12 months. At present, the patient’s best-corrected vision is 1/20. The left eye shows no signs of inflammation (Figure 1C) or *Acanthamoeba* infection (Figure 1F) using IVCM but there are a few superficial blood vessels in the peripheral cornea. There is also a large, central corneal scar obscuring the visual axis. The intraocular appearance is normal, and ultrasonography indicates a normal posterior segment. The patient is currently awaiting a corneal transplantation.

## Conclusions

This case emphasizes the need to suspect *Acanthamoeba* infection in soft contact lens wearers who present with progressive ulcerative keratitis and in progressively worsening corneal ulcers that are unresponsive to the usual antimicrobial therapy. It is also important to consider the possibility of a bacterial coinfection with the *Acanthamoeba* species. The exact reason why this combined inflammation occurred in our patient is still unknown. However, inquiries should be made regarding the patient’s contact lens cleaning history and the potential contamination of the contact lenses. Previous reports have revealed the presence of *Pseudomonas aeruginosa* and *Acanthamoeba* in the microbiological cultures obtained from contact lenses [1,5,6]. In addition, previous studies have shown that almost 50% of *Acanthamoeba*-positive eyes had cultures that were positive for bacteria as well [2,7]. A bacterial infection capable of supporting amoebic growth might play an important role in the pathogenesis of AK [8]. In light of these observations, it is advisable to use real-time polymerase chain reaction or IVCM in the early detection and treatment of AK. This case emphasizes the importance of suspecting *Acanthamoeba* infection in at-risk patients.

## Consent section

Written informed consent was obtained from the patient for publication of this case report and any accompanying images. A copy of the written consent is available for review by the Editor of this journal.

## Competing interests

The authors declare that they have no competing interests.

## Authors’ contributions

JH and JJ contributed to the conception and design, and the acquisition, analysis, and interpretation of the data. ZL, JX, and XS have been involved in the drafting and critical revision of the manuscript for important intellectual content. All the authors have given final approval of the version to be published.
